# Necrology

**Published:** 1897-08

**Authors:** 


### NECROLOGY.

Dr. W. D. Farnum, of Rockland, Me., a graduate of the American Veterinary College, class of 1889, died suddenly at Rockland, Me., falling dead from a sulky while driving a trotting horse, of which he was owner, in a race on July 5, 1897.

